# Distribution, Host-Seeking Phenology, and Host and Habitat Associations of *Haemaphysalis longicornis* Ticks, Staten Island, New York, USA

**DOI:** 10.3201/eid2504.181541

**Published:** 2019-04

**Authors:** Danielle M. Tufts, Meredith C. VanAcker, Maria P. Fernandez, Anthony DeNicola, Andrea Egizi, Maria A. Diuk-Wasser

**Affiliations:** Columbia University, New York, New York, USA (D.M. Tufts, M.C. VanAcker, M.P. Fernandez, M.A. Diuk-Wasser);; White Buffalo Inc., Moodus, Connecticut, USA (A. DeNicola);; Rutgers University, New Brunswick, New Jersey, USA (A. Egizi);; Monmouth County Mosquito Control Division, Tinton Falls, New Jersey, USA (A. Egizi)

**Keywords:** Haemaphysalis longicornis, Asian longhorned tick, ticks, distribution, host-seeking phenology, habitat associations, bacteria, vector-borne infections, zoonoses, Staten Island, New York, United States

## Abstract

*Haemaphysalis longicornis*, an invasive Ixodid tick, was recently reported in the eastern United States. The emergence of these ticks represents a potential threat for livestock, wildlife, and human health. We describe the distribution, host-seeking phenology, and host and habitat associations of these ticks on Staten Island, New York, a borough of New York City.

The invasive Asian longhorned tick, *Haemaphysalis longicornis*, is rapidly becoming an agricultural and epidemiologic concern in the United States. Native to eastern Asia, this tick is a major pest of domestic livestock throughout its invasive range in Australia, New Zealand, and surrounding islands (*1*,*2*) and also parasitizes wildlife and humans (*2*,*3*). The Asian longhorned tick is a vector of various pathogens infectious to humans (*3*,*4*), including severe fever with thrombocytopenia syndrome virus in China, which can be transmitted transstadially and transovarially to other ticks and mice (*5*). It is unknown whether these ticks also transmit pathogens endemic to the United States, or could contribute to the galactose-α-1,3-galactose meat allergy currently associated with *Amblyomma* and *Ixodes* tick species (*6*).

Although *H. longicornis* ticks have been detected by the US Department of Agriculture on quarantined horses and livestock for several years, it only recently became a health concern when high densities were found feeding on a nonimported domestic sheep in New Jersey (*7*) and in field collections in New York, New York (W. Bajwa, pers. comm.). These ticks have also been detected in eastern and southern US states including New York, Pennsylvania, Connecticut, New Jersey, Maryland, West Virginia, Virginia, North Carolina, and Arkansas (*3*).

Rapid emergence of *H. longicornis* ticks might be facilitated by their ability to reproduce parthenogenetically (*3*,*4*,*7*,*8*) and tolerate a wide range of environmental temperatures (−2°C to 40°C), although they are most successful in moist, warm-temperate conditions (*9*). *H. longicornis* ticks are a 3-host tick, similar to other Ixodid ticks (*9*), and have variable phenology depending on latitude (*9*,*10*). The phenology of *H. longicornis* ticks in the United States has not been characterized.

*H. longicornis* ticks might feed on a wide range of mammalian and avian hosts, which enables rapid geographic expansion (*2*,*9*). In New Zealand, *H. longicornis* ticks prefer habitat with Dallas grass (*Paspalum dilatatum*) and rushes (*Juncus* spp.) (*9*), plants abundant throughout the Midwestern and southern United States that could provide suitable habitat. In New Jersey, *H. longicornis* ticks were found in areas with unmowed grass (*7*), suggesting that these ticks might occupy wider habitat ranges than *Amblyomma* and *Ixodes* ticks.

We aimed to assess the ability of *H. longicornis* ticks to establish in the United States, their potential to acquire endemic human pathogens, and possible exposure risk to humans. We investigated the questing phenology and host and habitat associations in public parks and peridomestic environments of an emerging population of *H. longicornis* ticks on Staten Island, New York, during 2017–2018.

## The Study

All protocols and procedures were approved by the Institutional Review Board of Columbia University (protocol AAAR3750) and an Institutional Animal Care and Use Committee (protocols AC-AAAX4454 and AC-AAAS6470). Sampling efforts on public lands began in June 2017 when 13 forest sites were surveyed for questing ticks. During June 30–August 10, 2017, we removed avian-derived ticks from mist-netted birds at Freshkills Park on Staten Island. During June 3–August 24, 2018, we surveyed 24 forest and grassland sites for questing ticks and 8 forested grid sites for questing and mammal-derived ticks. Questing ticks were obtained by dragging a 1-m^2^ corduroy cloth over the leaf litter, which was checked every 10 m or 20 m and all attached ticks were removed. White-footed mice (*Peromyscus leucopus*) were trapped biweekly for 2 consecutive nights in Sherman live traps (https://www.shermantraps.com) separated by 10 m in 10 × 5 trap grids.

During August 5– 21, 2018, we anesthetized 16 male white-tailed deer (*Odocoileus virginianus*) and groomed them for ticks from 4 locations: College of Staten Island, Clay Pit Ponds, Mount Loretto State Forest and Freshkills Park. Mount Loretto State Forest, and Freshkills Park are dominated by grassland and wetland ecosystems, and Clay Pit Ponds is characterized by dense forest. College of Staten Island has manicured landscaping with grass and forest (separated by fencing) along the perimeter. While deer were anesthetized, we checked antlers, ears/head, front legs, hind legs, and body for ticks. Each animal was screened for 15 min to minimize time spent under anesthesia. Ticks were removed with forceps and stored in 100% ethanol for later identification.

During June 6–July 13, 2018, tick sampling was conducted on residential properties, which were selected by using a random cluster sampling strategy within areas previously identified as high risk given their proximity to parks (within 100 m). Houses were visited once, and questing ticks were collected along property edges by using the same method as in public parks.

**Figure 1:**
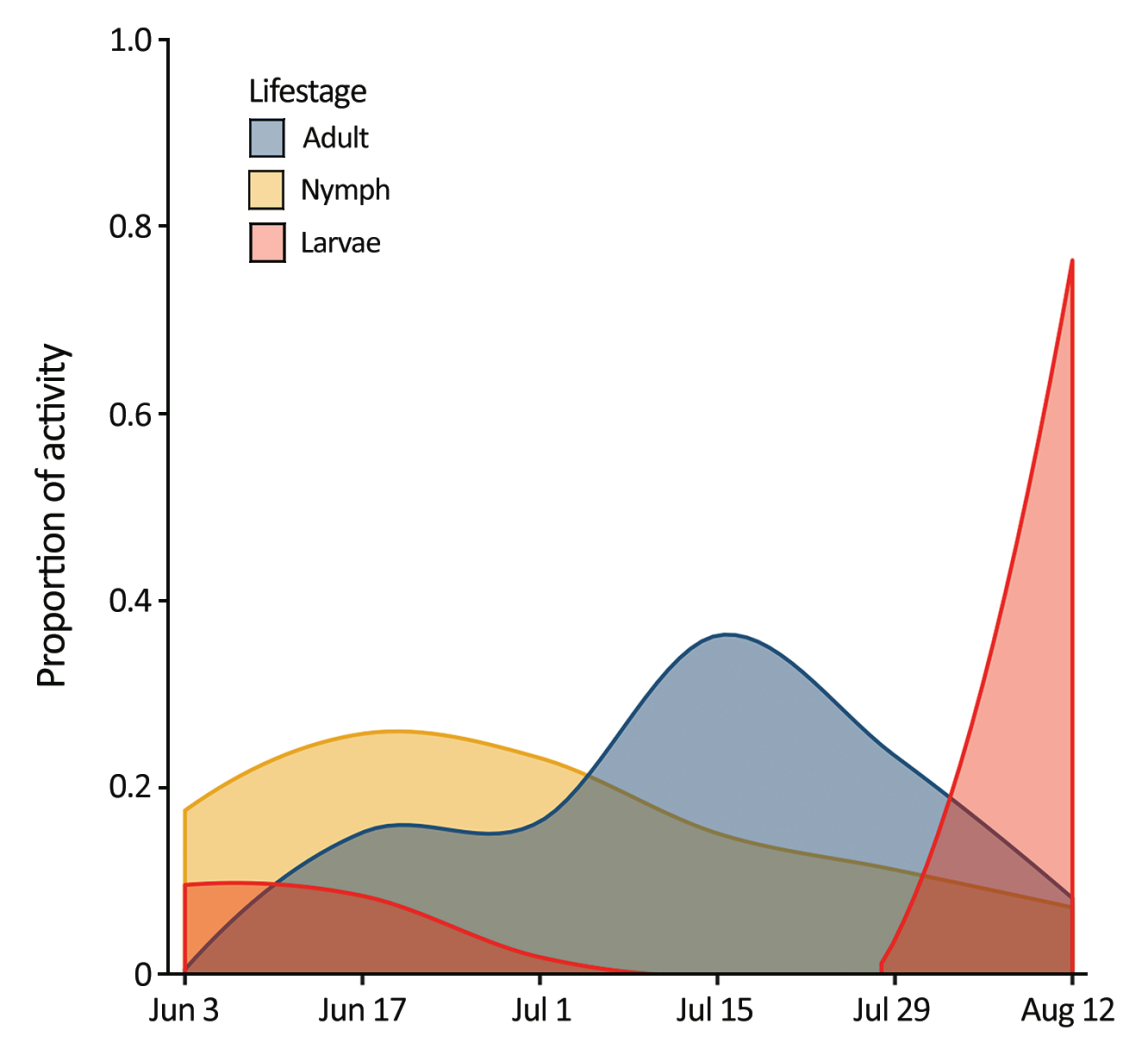
Seasonal activity of *Haemaphysalis longicornis* ticks (adults, nymphs, and larvae), Staten Island, New York, USA. Questing ticks were pooled by 2-week collection sessions during June 3–August 23, 2018.

We found questing *H. longicornis* ticks at 7 of 13 parks surveyed in 2017 and 16 of 32 parks surveyed in 2018 (Table 1). Adult ticks were most active in late July and nymphs were active from mid-June to mid-July, similar to findings in South Korea (*11*). Larvae showed highest proportional activity in late August (Figure 1). We identified ticks primarily by morphology; a representative sample of specimens from deer and drag collections (n = 63) were confirmed as *H. longicornis* ticks by DNA barcoding using the cytochrome c oxidase I locus (*12*).

**Table 1:** Sites surveyed for *Haemaphysalis longicornis* ticks, Staten Island, New York, USA, 2017 and 2018*

Year and location	Surveyed	Habitat	Density/1,000 m^2^*	Adult	Nymph	Larvae
2017						
Bloomingdale Park	Questing	Forest	3.1 (2)	0	5	0
Blue Heron Park	Questing	Forest	0.9 (2)	0	0	1
Clay Pit Ponds State Park Preserve	Questing	Forest	61.5 (2)	5	89	0
Clove Lakes Park	Questing	Forest	0.0 (2)	0	0	0
Conference House Park	Questing	Forest	7.4 (2)	7	78	0
Freshkills Park	Questing; host	Grassland	0.0 (2)	0	0	0
Great Kills Park	Questing	Grassland	0.0 (2)	0	0	0
High Rock Park	Questing	Forest	0.0 (2)	0	0	0
Latourette Park	Questing	Forest	1.3 (3)	0	3	0
Lemon Creek Park	Questing	Grassland	0.0 (2)	0	0	0
Silver Lake Park	Questing	Grassland	0.3 (2)	0	1	0
Willowbrook Park	Questing	Forest	0.8 (2)	0	1	0
Wolfe’s Pond Park	Questing	Forest	0.0 (2)	0	0	0
2018						
Arden Woods Park	Questing; host	Forest	2.7 (6)	2	4	2
Bloomingdale Park	Questing	Forest	5.9 (3)	0	13	0
Blueberry Park	Questing	Forest	0.0 (1)	0	0	0
Blue Heron Park	Questing; host	Forest	0.3 (6)	0	1	0
Bunker Pond Park	Questing	Forest	1.4 (1)	1	0	0
Clay Pit Ponds State Park Preserve	Questing; host	Forest	146.2 (3)	26	280	70
Clove Lakes Park	Questing; host	Forest	0.0 (6)	0	0	0
College of Staten Island	Questing; host	Grassland	676.0 (1)	0	0	169
Conference House Park	Questing; host	Forest	509.7 (6)	128	1,058	343
Deere Park	Questing	Forest	0.0 (1)	0	0	0
Freshkills Park	Questing; host	Grassland	11.0 (1)	4	7	0
Goodhue Park	Questing; host	Forest	0.3 (6)	0	1	0
Great Kills Park	Questing	Grassland	0.0 (1)	0	0	0
High Rock Park	Questing	Forest	3.3 (1)	0	2	0
Hybrid Oak Woods Park	Questing	Forest	13.6 (3)	6	18	1
Ingram Woods Park	Questing	Forest	0.0 (1)	0	0	0
Jones Woods Park	Questing	Forest	0.0 (1)	0	0	0
King Fisher Park	Questing; host	Forest	0.0 (6)	0	0	0
Latourette Park	Questing; host	Forest	5.3 (6)	3	3	10
Lemon Creek Park	Questing	Grassland	0.0 (1)	0	0	0
Midland Field Park	Questing	Forest	0.0 (1)	0	0	0
Miller Field Park	Questing	Forest	0.0 (1)	0	0	0
Mount Loretto State Forest	Questing; host	Grassland	675.0 (1)	2	1	78
Ocean Breeze Park	Questing	Grassland	0.0 (1)	0	0	0
Reed's Basket Willow Swamp Park	Questing	Forest	0.0 (2)	0	0	0
Seidenberg Park	Questing	Forest	0.0 (1)	0	0	0
Silver Lake Park	Questing	Grassland	0.0 (1)	0	0	0
﻿ Wegener Park	Questing	Forest	0.0 (1)	0	0	0
Westwood Park	Questing	Forest	0.7 (1)	1	0	0
Willowbrook Park	Questing; host	Forest	1.0 (6)	0	2	1
Wolfe's Pond Park	Questing	Forest	0.0 (1)	0	0	0
Woodhull Park	Questing	Forest	1.7 (1)	0	1	0

*Adult, nymph, and larval counts include questing ticks only. Values in parentheses indicate no. visits per site.

In 2017, we processed 39 birds: 11 American robins (*Turdus migratoriu*), 1 common yellowthroat (*Geothlypis trichas*), 20 gray catbirds (*Dumetella carolinensis*), 2 house wrens (*Troglodytes aedon*), 1 indigo bunting (*Passerina cyanea*), 3 northern cardinals (*Cardinalis cardinalis*), and 1 American yellow warbler (*Setophaga petechia*). We found no *H. longicornis* ticks of any life stage. We found no *H. longicornis* ticks on 87 uniquely tagged *P. leucopus* mice (190 individual captures), 2 eastern chipmunks (*Tamias striatus*), 14 northern short-tailed shrews (*Blarina brevicauda*), and 1 brown rat (*Rattus norvegicus*). All life stages were recovered from deer at all 4 locations, and larvae were the most prevalent stage collected (Table 2). These findings indicate that immature stages of these ticks might feed exclusively on white-tailed deer or unsampled medium-sized animals. The high proportion of larvae on deer was probably caused by sampling during peak larval activity.

**Table 2:** Number of *Haemaphysalis longicornis* ticks removed from 16 deer on Staten Island, New York, USA

Location*	Adult	Nymph	Larvae	Total
City University of New York College of Staten Island, n = 10	7	1	193	201
Clay Pit Ponds State Park Reserve, n = 3	56	63	217	336
Mount Loretto State Forest, n = 2	90	4	63	157
Freshkills Park, n = 1	15	6	140	161
Total, n = 16	168	74	613	855

*Values indicate no. deer screened at each location.

**Figure 2:**
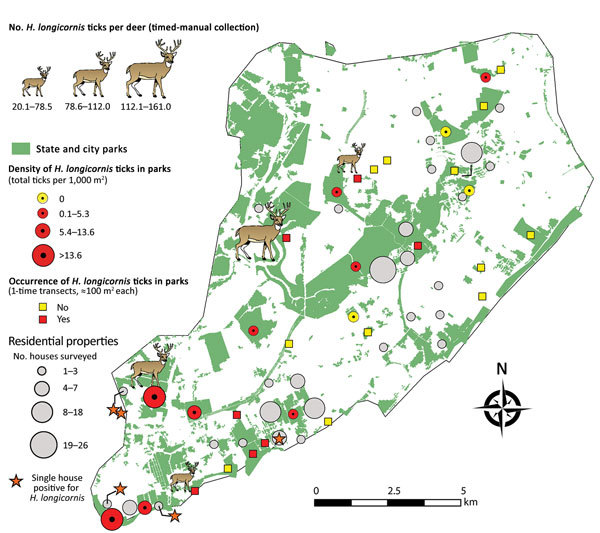
Sampling site locations and number of *Haemaphysalis longicornis* ticks collected on deer, in parks (grids and transects), and during household visits on Staten Island, New York, USA.

We surveyed 135 residential properties (average size 1,455 m^2^) (total visited = 505), of which 80% were located adjacent to parks in south and central Staten Island (Figure 2). Ticks were present at 34.1% of inspected properties. *H. longicornis* nymphs (n = 16) were found at 5 properties (in tall grass and shaded lawns), all located in the southern section of the island; no other life stages were found.

## Conclusions

The ability of *H. longicornis* ticks to feed on a wide range of domestic and wildlife hosts, reproduce asexually, and survive various environmental conditions likely contributed to their establishment throughout the eastern United States. All 3 life stages were found feeding on white-tailed deer in August, together with 2 other established tick species (*A. americanum* and *I. scapularis*). Pathogen transmission by co-feeding has been documented in field and laboratory studies (*13*,*14*) and could be a potential mechanism for *H. longicornis* ticks acquiring pathogens when feeding alongside infected *A. americanum* or *I. scapularis* ticks.

We found no *H. longicornis* ticks on *P. leucopus* mice or avian hosts even in sites with high densities of questing ticks, limiting the potential for this tick to acquire human pathogens. In New Zealand, immature life stages were commonly found on hares and goats, and adults were frequently found on larger mammals; the brown hare (*Lepus europaeus*) was touted as a major disseminator (*9*,*15*). These findings indicate the need for extensive sampling of other mammalian hosts. Finding *H. longicornis* ticks on residential properties is a human health concern because its potential as a vector of human pathogens in the United States is unknown. The combination of suitable habitat types, a plethora of host species, and high humidity make most regions of the United States suitable for *H. longicornis* tick establishment and proliferation.
